# Molecular characterization and identification of proteins regulated by Hfq in *Neisseria meningitidis*

**DOI:** 10.1111/j.1574-6968.2009.01568.x

**Published:** 2009-04-03

**Authors:** Yvonne Pannekoek, Robert Huis in ‘t Veld, Carla Th P Hopman, Ankie AJ Langerak, Dave Speijer, Arie van der Ende

**Affiliations:** 1 Department of Medical Microbiology, Center for Infection and Immunity Amsterdam (CINIMA), Academic Medical CenterAmsterdam, The Netherlands; 2 Clinical Proteomics Facility, Department of Medical Biochemistry, Academic Medical CenterAmsterdam, The Netherlands

**Keywords:** *Neisseria meningitidis*, Hfq, proteomics, riboregulation

## Abstract

Hfq is a highly conserved pleiotropically acting prokaryotic RNA-binding protein involved in the post-transcriptional regulation of many stress-responsive genes by small RNAs. In this study, we show that Hfq of the strictly human pathogen *Neisseria meningitidis* is involved in the regulation of expression of components involved in general metabolic pathways, iron metabolism and virulence. A meningococcal *hfq* deletion strain (H44/76Δ*hfq*) is impaired in growth in nutrient-rich media and does not grow at all in nutrient-limiting medium. The growth defect was complemented by expression of *hfq* in *trans*. Using proteomics, the expression of 28 proteins was found to be significantly affected upon deletion of *hfq*. Of these, 20 proteins are involved in general metabolism, among them seven iron-responsive genes. Two proteins (PilE, TspA) are involved in adherence to human cells, a step crucial for the onset of disease. One of the differentially expressed proteins, GdhA, was identified as an essential virulence factor for establishment of sepsis in an animal model, studied earlier. These results show that in *N. meningitidis* Hfq is involved in the regulation of a variety of components contributing to the survival and establishment of meningococcal disease.

## Introduction

A prokaryotic riboregulated network, in which small RNAs (sRNAs), in conjunction with specific proteins, regulate the translation and/or the decay of mRNAs has recently been discovered (Majdalani *et al.*, 2005). These regulatory events commonly require the action of the Sm-like protein Hfq. Hfq is a strikingly conserved pleiotropically acting RNA-binding protein, facilitating base pairing between sRNA and mRNA, which in general may either decrease ribosome binding or unmask the RNAseE cleavage site, leading to mRNA decay, or may improve ribosome binding, leading to mRNA stability (Valentin-Hansen *et al.*, 2004). Recent studies have shown that Hfq extensively impacts bacterial physiology, including control of virulence factors. Null mutants of *hfq* of a variety of pathogens are highly attenuated in animal models (Geissmann & Touati, 2004; Valentin-Hansen *et al.*, 2004; Brennan & Link, 2007; Sittka *et al.*, 2007).

The strictly human pathogen *Neisseria meningitidis* causes septicemia and meningitis, a life-threatening disease, especially in childhood, and is a serious public health problem worldwide (de Souza & Seguro, 2008). This pathogen possesses a variety of genes involved in the adaptation to the different environments encountered in the host, including iron depletion (Grifantini *et al.*, 2003; Delany *et al.*, 2004; Basler *et al.*, 2006). All four available completely sequenced genomes of *N. meningitidis* contain a gene with significant homology to *hfq* (Parkhill *et al.*, 2000; Tettelin *et al.*, 2000; Bentley *et al.*, 2007; Peng *et al.*, 2008). *Hfq* of *N. meningitidis* was identified as an essential gene for the onset of septicemia using signature-tagged transposon-mutated meningococci in an infant rat model (Sun *et al.*, 2000). This observation strongly suggests that Hfq and genes under control of Hfq in the meningococcus are of importance for establishing disease. To identify which genes of *N. meningitidis* are regulated by Hfq, we constructed an *hfq* knock-out strain and used a proteomic approach to identify proteins whose expression is under control of Hfq.

## Materials and methods

### Bacterial strains and culture conditions

*Neisseria meningitidis* strain H44/76, B: P1.7,16: F3-3: ST-32 (cc32), is closely related to the sequenced serogroup B strain MC58 and belongs to the same clonal complex (van der Ende *et al.*, 1999). Meningococci were cultured in tryptic soy broth (TSB) (BD), GC broth or on GC plates (Difco) supplemented with 1% (v/v) Vitox (Oxoid) at 37 °C in a humidified atmosphere of 5% CO_2_ (van der Ende *et al.*, 1999). If appropriate, plates or broth were supplemented with erythromycin (5 μg mL^−1^) and/or chloramphenicol (25 μg mL^−1^). Growth was monitored by measuring the OD_600 nm_ of cultures at regular time intervals. Growth experiments were repeated five times.

### Construction of an *hfq* knock-out mutant of *N. meningitidis*

An *N. meningitidis* H44/76 *hfq* knock-out mutant (H44/76Δ*hfq*) was constructed using the PCR-ligation-PCR method (Ali & Steinkasserer, 1995; van der Ende *et al.*, 1999). PCR products were generated with primer pairs ABHfq1/ABHfq2 and ABHfq3/ABHfq4, ligated and the ligation product was reamplified with the primer pair ABHfq1/ABHfq4. The resulting PCR product was cloned into pCR2.1 (Invitrogen). The EcoRI-digested erythromycin resistance cassette from pAErmC' (Zhou & Apicella, 1996) was introduced into the created unique MfeI restriction site, yielding plasmid pHfq10. H44/76Δ*hfq* was generated by natural transformation of strain H44/76 with pHfq10 and selection for erythromycin resistance. Replacement of *hfq* (NMB0748) by the erythromycin cassette was confirmed by PCR with combinations of primers JP19-20, JP22, ABHfq1 and ABHfq4. Oligonucleotides are listed in Table 1.

**Table 1:** Oligonucleotides used in this study

Name	Sequence 5′–3′	GenBank accession number	Location
ABHfq1	CCGGCGGCATGGGCGCAT	AE002098.2*	780118..780135
ABHfq2	TTTTAACTCCGTTATTATGATTGTG	AE002098.2	779576..779600
ABHfq3	ACAATTGAATCCGCACGAAGCATGA	AE002098.2	779281..779264
ABHfq4	CAGGTTTTCATGTCCGTCCA	AE002098.2	778947..778966
ALHfq11	GGGGCATATGACAGCTAAAGGACAA	AE002098.2	779570..779556
ALHfq12	CCCCTCATGATTCGGCAGGCTGCTGGAC	AE002098.2	779283..779300
ALHfq13	CAGAGAAGGCATGTGGAACA	AE002098.2	779891..779872
ALHfq14	TCAGGTTGAGTCTTTCGATCA	AE002098.2	779469..779449
ALHfq15	TGGGTGACGGAAGTGTTTCT	AE002098.2	779413..779432
ALHfq16	GATGTCGAATGCCCACACTT	AE002098.2	779037..779056
NMB0747F	ATGTTATTGCAAAACATCCTTC	AE002098.2	779195..779174
NMB0747R	TTATTTTTGACGCAGTTTTTCA	AE002098.2	778629..778650
JP19	TAAATACAAAACGCTCATTGGC	M17990.1†	2086..2107
JP20	ACCTCTTTACTAATTCAAGGGT	M17990.1	1813..1833
JP22	AAATCGTCAATTCCTGCATGTT	M17990.1	2329..2350

Genomic localization according to

* Tettelin *et al.* (2000), GenBank AE002098.2 and

† Projan *et al.* (1987), GenBank M17990.1.

### Complementation of H44/76Δ*hfq*

To complement the *hfq* deletion, *hfq* from strain H44/76 was amplified with the primer pair ALHfq11/ALHfq12, containing NdeI and RcaI restriction sites, respectively. The resulting PCR product and shuttle vector pEN11-pldA (Bos *et al.*, 2005) were digested with NdeI and RcaI, and the PCR product was cloned into NdeI–RcaI-predigested pEN11-pldA and transformed into *Escherichia coli* TOP10F' (Invitrogen). Chloramphenicol-resistant colonies were checked by colony PCR and sequencing, using universal M13 primers. Plasmid DNA of a clone containing a complete intact *hfq*-coding region gene (pEN11-*hfq*2) was isolated and used to transform H44/76Δ*hfq*. The expression of *hfq* was induced by addition of IPTG to the culture medium to a final concentration of 1 mM. The DNA sequence of *hfq* of H44/76 was deposited into GenBank (FJ606876).

### Reverse transcriptase (RT)-PCR

RNA was isolated using the Rneasy® Midi Kit (Qiagen). RT-PCR was performed using SuperScriptIII (Invitrogen). Primer pairs ALHfq13/ALHfq15, ALHfq13/ALHfq16 and ALHfq14/ALHfq16 were used to investigate whether NMB0747-NMB748-NMB749 is transcribed as a polycistronic operon and primer pair ALNMB0747F/ALNMB0747R to investigate the transcription of NMB0747 in H44/76Δ*hfq*.

### Cell fractionation

Meningococci were grown in broth until OD_600 nm_=0.6–0.8, harvested by centrifugation (10 min at 3000 ***g***) and resuspended in 50 mM Tris-HCl (pH 7.8). Of the remaining culture medium, blebs were removed by centrifugation (100 000 ***g***, 1 h, 4 °C). The supernatant thus obtained was used as a source of secreted proteins. Meningococcal cells were disrupted by sonication (Branson B15 Sonifier, 50 W, 10 min, 50% duty cycle, 4 °C), followed by centrifugation (3000 ***g***, 10 min, 4 °C). The supernatant was centrifuged (28 000 ***g***, 30 min, 4 °C) and pellets, containing the cell envelops (inner and outermembranes), were resuspended in 2 mM Tris-HCL (pH 6.8) containing 1% sodium lauroyl sarcosinate and incubated overnight at 4 °C to dissolve inner membranes. The outer membrane fraction was then obtained by centrifugation (100 000 ***g***, 2 h, 4 °C) and dissolved overnight in 200 μL 2 mM Tris-HCl (pH 6.8) at 4 °C. All fractions were stored at −20 °C. Protein concentrations were determined by Protein Assay (Bio-Rad).

### One-dimensional (1D) sodium dodecyl sulfate polyacrylamide gel electrophoresis (SDS-PAGE)

Proteins were resolved by SDS-PAGE (Laemmli, 1970). Gels (12% or 25%) were stained with the PageBlue kit (Fermentas), washed in MilliQ water and stored in 1% acetic acid at 4 °C until the bands of interest were excised for further analysis.

### 2D gel electrophoresis and matrix-assisted laser deionization/ionization time of flight (MALDI-TOF) MS

Samples were dissolved in 2D sample buffer (7.7 M urea, 2.2 M thiourea, 30 mM Tris-HCl, pH 8.5, 4% CHAPS and a trace of bromophenol blue); 1.25 μL DeStreak solution (GE Healthcare) and 2.5 μL immobilized pH gradient (IPG) buffer, pH 4–7 (GE Healthcare) were added. First dimension IPG-strips (Immobiline DryStrip, pH 4–7, GE Healthcare) were applied on top of the sample solution, covered with oil and incubated overnight at room temperature (RT). Isoelectric focusing was performed on the Protean IEF cell (Bio-Rad) basically with gradually increasing voltage up to 3500 V according to the standard GE Healthcare protocol. After IEF, strips were incubated in 1 mL equilibration buffer (50 mM Tris-HCl, pH 8.8, 6 M urea, 30% glycerol, 2% SDS and a trace of bromophenol blue) with 10 mg dithiothreitol added for 20 min on a shaker at RT. Strips were transferred to 1 mL equilibration buffer with 25 mg iodoacetamide and again incubated for 20 min. The IPG strip was transferred from the tube to the top of a 12% SDS-PAGE gel and the gel was covered with a warm solution of 1% low-melting point agarose, 15% glycerol and a trace of bromophenol blue in Laemmli buffer. Electrophoresis was conducted at 200 V. Gels were stained with PageBlue, washed in MilliQ water and stored in 1% acetic acid at 4 °C until spots of interest were excised for further analysis. MALDI-TOF MS was carried out as described previously (Pannekoek *et al.*, 2005). All included protein assignments are unambiguous. Monoisotopic peaks in the peptide mass fingerprint spectrum were searched against the complete nonredundant database of all organisms (MSDB at MASCOT). Only in case of a significant MOWSE score of a meningococcal protein as the top hit was the identification considered reliable (pIs and MWs were also not restricted in the search and were found to match). We only analyzed those proteins whose expression was reproducibly and markedly altered (‘on–off’ proteins) when comparing wt, H44/76Δ*hfq* and pEN11-*hfq*2 complemented cells (in both 1D and 2D gels). This approach guarantees that the protein identified is indeed the one under Hfq control.

## Results

### Hfq is conserved among neisserial species and part of a polycistronic operon

**Fig. 1:**
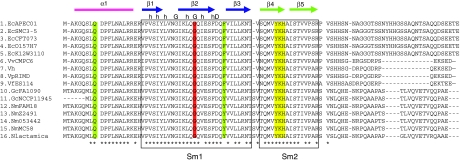
Amino acid sequence alignment of Hfq proteins of *Escherichia coli* serotypes, *Vibrio* species, *Neisseria meningitidis, Neisseria gonorrhoeae* strains and *Neisseria lactamica*. The secondary structural elements of Hfq protein are shown above the alignment with the N-terminal helix α1 in cyan. The Sm1 and Sm2 motif regions are boxed. The sole conserved residue Gly34 is indicated in red. Highly conserved hydrophobic residues within the Sm1 region are indicated by a lower case h, and the two highly conserved glycine and aspartic acid residues within the Sm1 motif are indicated by G and D, respectively. The absolutely conserved glutamine of helix α1 that is important for base recognition and the highly conserved tyrosine (or phenylalanine) are indicated in green. Within the Sm2 region, the ‘Hfq Sm2 motif YKH’ is colored yellow. Other conserved residues are indicated by an asterisk. Note the minimal sequence variation between Hfq of *E. coli* serotypes (lines 1–5), *Vibrio vulnificus* (line 6), *Vibrio harveyi* (line 7), *Vibrio parahaemolyticus* (line 8), *Vibrio fischeri* (line 9), *N. gonorrhoeae* strains (lines 10 and 11), *N. meningitidis* strains (lines 12-15) and *N. lactamica* (line 16) (adapted from Schumacher *et al.*, 2002).

The complete genome sequences of four meningococcal, two strains of the closely related human pathogen *Neisseria gonorrhoeae* and one commensal neisserial species (*Neisseria lactamica*) are known (Parkhill *et al.*, 2000; Tettelin *et al.*, 2000; Bentley *et al.*, 2007; Chung *et al.*, 2008; Peng *et al.*, 2008). The amino acid sequence of Hfq of the meningococcal strain used in this study (H44/76) is identical to the Hfq sequence of meningococcal strain FAM18 and 98% identical to the sequences of the other three meningococcal strains. In addition, the sequence of H44/76 is 98% identical to gonococcal Hfq and 95% identical to the Hfq sequence of *N. lactamica*. The H44/76 neisserial Hfq amino acid sequence shows 63% and 60% identities to Hfq proteins of *E. coli* and *Vibrio* spp., respectively (Fig. 1). Of importance, hardly any amino acid substitutions in the meningococcal Hfq sequence, compared with those of *E. coli* and *Vibro* spp., are observed in highly conserved regions of the protein shown to be important for functionality (Fig. 1) (Schumacher *et al.*, 2002).

**Fig. 2:**
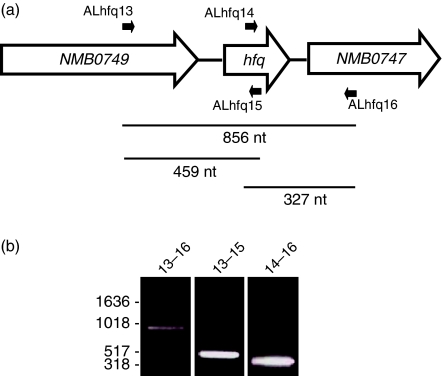
Transcriptional analysis of the Hfq operon. (a) Schematic representation of the Hfq polycistronic operon. Primers used in RT-PCR are indicated in black arrows. The size of the calculated RT-PCR products is indicated below the black lines. (b) RT-PCR results. Products obtained by RT-PCR were separated on agarose gel. Numbers on the left represent marker sizes; primer pairs used in the RT-PCR are indicated above the lanes. Reactions in which the addition of reverse transcriptase was omitted did not yield any products (not shown).

The genetic organization of the chromosomal locus of *hfq* among all, except one (*N. gonorrhoeae* FA1090), neisserial strains investigated is also conserved. In neisserial spp. the *hfq* gene is preceded by a gene encoding d-alanyl-d-alanine endopeptidase (NMB0749, *pbp7*). Eighty-four basepairs downstream of neisserial *hfq*, an ORF encoding a conserved hypothetical protein is found (NMB0747, Fig. 2a). To determine whether neisserial *hfq* was transcriptionally linked to either of the two flanking genes, RT-PCR analysis was performed, templated by total RNA from meningococcal strain H44/76 using primers annealing to *pbp7* and the downstream gene. This RT-PCR yielded a product of *c*. 800 bp, indicating that *hfq* is transcriptionally linked to *pbp7* and NMB0747 (Fig. 2b).

### Hfq is required for optimal growth of *N. meningitidis*

**Fig. 3:**
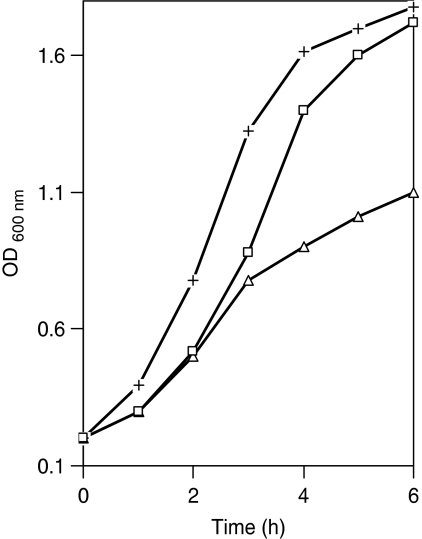
Growth kinetics of H44/76Δ*hfq* and complementation of the growth defect by expression of neisserial *hfq* in *trans*. Growth of the strains at 37°C was followed by measuring the density of the cultures at intervals. +, H44/76 wt strain; ▵, H44/76Δ*hfq*; □, H44/76Δ*hfq*+pEN11-*hfq*2 (induced by IPTG). A representative experiment is shown.

An H44/76Δ*hfq* strain of *N. meningitidis* was created by complete gene replacement of *hfq* with an erythromycin resistance cassette. Upon examination of the growth characteristics of the H44/76Δ*hfq* strain, it was observed that this strain formed very tiny colonies after overnight growth on rich solid media, compared with the wild-type (wt) strain. In addition, the strain did not grow in TSB, a relatively nutrient-poor broth, and exhibited a growth deficiency in GC broth, compared with the wt strain (Fig. 3). As the erythromycin resistance cassette used here does not contain a terminator, transcription of NMB0747 should be unaffected. This was confirmed by RT-PCR (not shown). In addition, the expression of *hfq* in *trans* in GC broth clearly restored growth (Fig. 3).

### Identification of proteins differentially expressed in H44/76 wt as compared with the H44/76Δ*hfq* strain

**Fig. 4:**
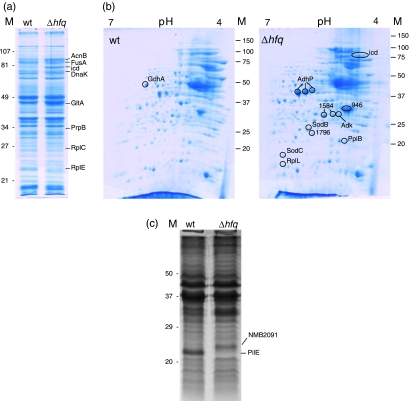
Proteins differentially regulated in H44/76Δ*hfq*. (a) 1D analysis of protein patterns of a whole-cell lysate of wt and H44/76Δ*hfq* cells. (b) 2D analysis of protein patterns of whole-cell lysates of wt (left panel) and H44/76Δ*hfq* cells (right panel). (c) 1D analysis of cell envelops of wt and H44/76Δ*hfq* cells. Differentially expressed proteins comparing wt and H44/76Δ*hfq* cells are indicated. Whole-cell lysates were run on 12% gels, and cell envelops on 25% gels.

To identify genes whose expression is controlled by Hfq, protein patterns of the wt strain, the H44/76Δ*hfq* strain and the complemented strain were compared by 1D and 2D gels. Only those proteins, whose differential regulation was confirmed in at least two independent experiments and whose expression was turned to wt strain levels in the complemented strain were considered to be truly differentially regulated. Proteomic analyses of patterns of whole-cell lysates, cytoplasm, cell envelops, outer membranes and growth medium (secreted proteins) showed that the expression of at least 28 proteins in *N. meningitidis* was affected by Hfq (Fig. 4). Of these 28 proteins, 23 were upregulated in the H44/76Δ*hfq* strain while the other five proteins (PilE, RplE, GdhA, AtpA and AtpD) were downregulated in the absence of Hfq (Fig. 4, Table 2). The majority (*n*=19) of the differentially regulated proteins were identified in the whole-cell lysates and/or cytoplasmic fractions, eight proteins were identified in membrane fractions and one protein (isocitrate dehydrogenase: icd; NMB0920) was identified in both the whole-cell lysate as well as in the secreted protein fraction of the H44/76Δ*hfq* strain (Fig. 4a and b, Table 2).

**Table 2:** Differential regulated proteins in *H44/76*Δ*hfq*

Gene ID*	Name†	Location‡	Reg^−§^	Functions¶	Functional class∥	MW**	pI††	Figure‡‡
NMB0018	PilE	CE	−	Type IV pilus assembly protein PilE	Surface structure	18 072	9.6176	4c
NMB0138	FusA	W	+	Elongation factor G	Protein translation and modification	77 244	4.8184	4a
NMB0142	RplC	W	+	50S ribosomal protein L3	Ribosomal protein synthesis and modification	22 678	10.7606	4a
NMB0154	RplE	W	−	50S ribosomal protein L5	Ribosomal protein synthesis and modification	20 322	10.0609	4a
NMB0341	TspA§§	C	+	T-cell-stimulating protein A	Unknown	92 488	4.0561	NS
NMB0430	PrpB	W, C	+	Putative carboxyphosphonoenol pyruvate phosphonomutase	Carbohydrate metabolism	31 714	5.0352	4a
NMB0431	PrpC	C	+	Methylcitrate synthase	Carbohydrate metabolism	42 818	7.1147	NS
NMB0546	AdhP	W	+	Alcohol dehydrogenase (propanol preferring)	Fermentation	36 547	5.8432	4b
NMB0554¶¶	DnaK	W	+	Molecular chaperone	Environmental information processing	68 791	4.5862	4a
NMB0589	RplS	CE	+	50S ribosomal protein L19	Ribosomal protein synthesis and modification	13 767	11.0643	NS
NMB0634¶¶	FbpA	C	+	Iron(III) ABC transporter/major ferric iron-binding protein	Transport/binding proteins	35 827	10.1574	NS
NMB0791¶¶	PpiB	W	+	Peptidyl-prolyl *cis*–*trans* isomerase B (cyclophilin B)	Protein translation and modification	18 852	4.8602	4b
NMB0823	Adk	W	+	Adenylate kinase	Purine ribonucleotide biosynthesis	23 190	4.7879	4b
NMB0884¶¶	SodB	W, C	+	Superoxide dismutase, Fe–Mn family	Detoxification	21 892	6.1483	4b
NMB0920	icd	W, W, SP	+	Isocitrate dehydrogenase	Tricarboxylic acid cycle	80 163	5.6646	4a, 4b
NMB0946	–	W, OM	+	Peroxiredoxin 2 family protein	Unknown	26 912	4.5382	4b
NMB0954	GltA	W, C	+	Type II citrate synthase	Tricarboxylic acid cycle	48 121	6.7886	4a
NMB1320¶¶	RplI	W	+	50S ribosomal protein L9	Ribosomal protein synthesis and modification	15 747	7.5349	4b
NMB1388	Pgi-2	C	+	Glucose-6-phosphate isomerase	Glycolysis	62 084	6.5126	NS
NMB1398	SodC	W	+	Cu–Zn superoxide dismutase	Detoxification, periplasmic protein	19 520	6.6244	4b
NMB1572¶¶	AcnB	W, IM	+	Aconitate hydratase	Tricarboxylic acid cycle	92 715	5.2810	4a
NMB1584	–	W, C, C	+	3-Hydroxyacid dehydrogenase	Amino acid metabolism	30 378	5.1348	4b
NMB1710	GdhA	W	−	Glutamate dehydrogenase	Amino acid biosynthesis	48 490	6.1895	4b
NMB1796¶¶	–	W, C	+	Putative oxidoreductase	Metabolism	20 686	5.8148	4b
NMB1934	AtpD	IM	−	ATP synthase F1, β chain	ATP-proton motive force	50 391	4.7746	NS
NMB1936	AtpA	IM	−	ATP synthase F1, α chain	ATP-proton motive force	55 291	5.2754	NS
NMB2091	–	CE	+	Putative hemolysin	Membranes, lipoproteins and porins	21 745	10.2186	4c
NMB2129	ArgG	IM	+	Argininosuccinate synthase	Amino acid biosynthesis	49 664	4.9527	NS

* Gene identification according to Tettelin *et al.* (2000).

† Protein name according to Tettelin *et al.* (2000).

‡ Fraction in which the protein was identified: W, whole cell lysate; C, cytoplasm; CE, cell envelop; IM, innermembrane; OM, outermembrane; SP, secreted protein fraction.

§ Up- or downregulation in hfq knockout.

¶ Protein function according to KEGG (http://www.genome.jp/kegg).

∥ Functional classification according to Parkhill *et al.* (2000).

** Protein molecular weight according to JCVI-CMR (http://cmr.jcvi.org/cgi-bin/CMR/shared/Menu.cgi?menu=genome).

†† Isoelectric point according to JCVI-CMR (http://cmr.jcvi.org/cgi-bin/CMR/shared/Menu.cgi?menu=genome).

‡‡ Figure in which spot and/or band is identified. NS, not shown.

§§ Protein name according to Oldfield *et al.* (2007).

¶¶ Iron-responsive gene (Grifantini *et al.*, 2003; Delany *et al.*, 2004; Basler *et al.*, 2006).

Of the 28 proteins differentially expressed between the wt and the H44/76Δ*hfq* strain, 12 are functionally involved in general metabolism and eight are involved in ribosomal protein synthesis/modification, amino acid biosynthesis, protein translation and modification. Two proteins involved in detoxification (SodB and SodC), one iron(III) ABC transporter (FbpA) and one molecular chaperone (DnaK) were also identified. In addition, two proteins with a largely unknown function (encoded by NMB2091 and NMB0946) were also differentially expressed in the H44/76Δ*hfq* strain (Table 2). Of interest, in cell envelops of the H44/76Δ*hfq* strain, PilE, the structural subunit of the Type IV pili (Tfp) and a well-characterized virulence factor involved in adherence to cells and cell motility of meningococci (Nassif *et al.*, 1994; Oldfield *et al.*, 2007), was no longer detectable (Fig. 4c). In addition, a protein designated T-cell-stimulating protein A (TspA), also implicated in adherence of meningococci to cells (Oldfield *et al.*, 2007), was found to be upregulated in H44/76Δ*hfq*.

## Discussion

The Hfq protein is recognized as a major post-transcriptional regulator of bacterial gene expression participating as an RNA chaperone in numerous regulatory pathways (Valentin-Hansen *et al.*, 2004). In this study, we explored the Hfq regulon of the strictly human pathogen *N. meningitidis*.

Loss of Hfq function in the meningococcus (coding capacity *c*. 2200 genes) (Parkhill *et al.*, 2000) resulted in clear deregulation of *c*. 28 proteins (>1% of total coding capacity) as found by comparative proteomics. Using approaches comparable to ours, it was found that loss of Hfq function in *Salmonella enterica* serovar Typhimurium and *Pseudomonas aeruginosa* leads to deregulation of *c*. 2% and 5% of the total coding capacity, respectively (Sonnleitner *et al.*, 2003; Sittka *et al.*, 2007), and is thus comparable to what we found in meningococci.

The genetic organization of *hfq* of *N. meningitidis*, however, is different from that found in most other pathogens studied so far. For example, in *E. coli, S*. Typhimurium and *Listeria monocytogenes, hfq* is located downstream from *miaA*, which encodes a protein similar to tRNA isopentenylpyrophosphate transferase and upstream from *hflx*, encoding the putative GTP-binding protein (Christiansen *et al.*, 2004; Sittka *et al.*, 2007; Kulesus *et al.*, 2008). In these pathogens, *hfq* is usually cotranscribed with *miaA*, and transcription is terminated directly after *hfq* In *N. meningitidis hfq* is located downstream from *pbp7*, encoding d-alanyl-d-alanine-endopeptidase (penicillin-binding protein 7) and upstream of a hypothetical gene encoding a predicted rRNA methylase. We demonstrated by RT-PCR that meningococcal *hfq* is part of a polycistronic operon and cotranscribed with both of the flanking genes, a situation similar to what is found in *Moraxella catarrhalis*. In this pathogen, *hfq* is also cotranscribed with both of the flanking genes (*miaA* and *kpsF*, the latter encoding a predicted arabinose-5-phosphate isomerase) (Sittka *et al.*, 2007; Attia *et al.*, 2008; Kulesus *et al.*, 2008). Using an erythromycin cassette without a terminator, we avoided interfering with transcription of NMB0747, part of the polycistron and directly downstream of Hfq. The growth defect observed in meningococci upon deletion of *hfq* was substantially reversed upon expression of *hfq* in *trans*. This observation strongly suggests that Hfq plays an important role in the physiology of the meningococcus and that upon deletion metabolic processes are disturbed, resulting in a phenotype with impaired growth.

In some bacterial species, Hfq is also required for efficient translation of *rpoS* mRNA, encoding the general stress σ factor, and/or loss of Hfq results in activation of RpoE, the alternative σ factor mediating the response to envelope stress (Hengge-Aronis, 2002; Johansen *et al.*, 2006; Sittka *et al.*, 2007). Analyses of protein patterns on 1 and 2D gels showed that the expression of 28 genes of *N. meningitidis* is affected by Hfq, either directly or indirectly. The identified genes are most likely not under control of RpoS, because *N. meningitidis* does not possess an RpoS-like σ factor. Involvement of RpoE, however, cannot be ruled out, because the genome of *N. meningitidis* does contain a gene (NMB2144) encoding this alternative σ factor. The functionality of RpoE and its possible connection to the Hfq regulon in meningococci remains to be addressed.

Classification of the genes identified by comparative analyses shows that the majority of the encoded proteins belong to the functional classes of metabolism (*n*=12), ribosomal protein synthesis and modification, amino acid biosynthesis and protein translation and modification (*n*=8). Among these, the NADP-specific glutamate dehydrogenase (NADP-GDH, encoded by *gdhA*) was found to be downregulated in the absence of Hfq. This is an interesting observation, because *gdhA* has been shown to be an essential gene for systemic meningococcal infection in an infant rat model (Sun *et al.*, 2000) and its expression levels are the highest in strains belonging to the hypervirulent lineages ET-5 (serogroup B) and IV-1 (serogroup A) (Pagliarulo *et al.*, 2004). Downregulation of GdhA expression in the absence of Hfq suggests that Hfq might promote translation, for instance by stabilization of *gdhA* mRNA, and thus contribute to the virulence potential of *N. meningitidis*. Whether this occurs in conjunction with an sRNA remains to be addressed.

Of the 28 proteins identified, seven are encoded by genes whose expression is under control of iron and/or Fur (Grifantini *et al.*, 2003; Delany *et al.*, 2004; Basler *et al.*, 2006). Recently, an iron- and Fur-regulated sRNA (NrrF) was identified in *N. meningitidis* (Mellin *et al.*, 2007). This sRNA has been shown to repress expression of succinate dehydrogenase upon iron depletion and its interaction with the *sdhCDAB* transcript *in vitro* is enhanced in the presence of Hfq (Metruccio *et al.*, 2009). Although none of the identified proteins belong to the succinate dehydrogenase complex, seven other iron-responsive proteins were differentially expressed in the H44/76Δ*hfq* strain. This suggests that in *N. meningitidis* Hfq, possibly in conjunction with sRNAs, might also contribute to the fine-tuning of factors involved in iron homeostasis. One of the iron-responsive proteins, which is differentially expressed between wt and H44/76Δ*hfq*, is superoxide dismutase (SodB). In *E. coli*, SodB is known to be regulated by RyhB, a Fur-regulated Hfq-dependent sRNA (Masse & Gottesman, 2002). Also, in other pathogens, SodB expression is controlled by Hfq and an sRNA (Wilderman *et al.*, 2004; Mey *et al.*, 2005). It is tempting to speculate that in meningococci, SodB expression is also regulated by Hfq in conjunction with an sRNA. This sRNA remains to be identified.

We detected two proteins (PilE and TspA) known to be involved in the adherence of meningococci to human cells. Tfp-mediated adherence of meningococci to human cells leads to clumps of bacteria associated with microvillus-like structures on the surface of the cells (Nassif *et al.*, 1994; Wilderman *et al.*, 2004). This is followed by contact-dependent downregulation of pili and intimate adhesion, mediated by adhesins including TspA (Oldfield *et al.*, 2007). The absence of detectable PilE, the structural subunit of Tfp and the upregulation of TspA expression in H44/76Δ*hfq* suggests a role of Hfq in the meningococcal adherence strategies. The mechanisms responsible for these observations remain to be elucidated, but riboregulatory processes have previously been shown to be involved in Tfp assembly and cell motility of other bacterial pathogens (Sonnleitner *et al.*, 2003; Sittka *et al.*, 2007; Dienst *et al.*, 2008).
